# Embryological Aspects of Oocyte *In Vitro* Maturation

**Published:** 2013

**Authors:** Mir Mehrdad Farsi, Nematollah Kamali, Mohsen Pourghasem

**Affiliations:** 1 *Fatemeh Zahra Infertility and Health Reproductive Research Center, Babol University of Medical Sciences, Babol, Iran*.; 2 *Department of Anatomical Sciences, Babol University of Medical Sciences, Babol, Iran.*; 3 *Cellular and Molecular Biology Research Center (CMBRC), Babol University of Medical Sciences, Babol, Iran*.

**Keywords:** *In Vitro* Maturation, immature oocyte, follicle

## Abstract

*In Vitro* Maturation (IVM) is a method that immature oocytes in antral follicles are extracted and matured in laboratry conditions. This review has attempted to provide the current knowledge and recent findings in *in vitro* maturation of oocytes and highlights the most important factors involved in this process. The review is based on literature reports and the author’s experience. In IVM cycles, the time of administration of hCG is depending on the diameter of the largest follicle that has been determined to be about 10-12 mm to prevent the detrimental effect of dominant follicle (DF). Higher number of *in vivo* matured oocytes with dispersed cumulus cells (CC) pattern can be achieved by increasing the time of hCG injection up to 38 h. Growing of oocytes during the final hours of *in vitro* maturation has profound effect on the following outcome. Injection of IVM oocytes must be delayed at least 1 h after extrusion of the first polar body. IVM outcome shows that the pregnancy rate is low in pure immature oocytes except PCO(s) (Polycystic ovaries and Polycystic ovarian syndrome) cases. Furthermore, endometrial quality may have a crucial role in this respect after non hCG-triggered IVM. The formulation of different types of maturation media shows that they are generally supplemented with recombinant FSH and hCG. Taurine and calcium as unique components of blastocyst medium have been supposed to be valuable to IVM media. Pyruvate and adenosine triphosphate (ATP) and Epidermal Growth Factor (EGF) have been proposed as additives for maturation media. IVM is not a suitable treatment for women over 40 years. Different categories of patients could be candidate for IVM. Despite of old concept in low outcome and caution in IVM indications, innovative findings in this field have opened new windows in the treatment of patients.

Oocyte maturation that starts before birth and continues during folliculogenesis is a prolonged and complex process during which oocytes achieve developmental competence. Only a small number of these unique offered cells will have the chance to develop for probable fertilization. By harvesting and maturing outside the body (*in vitro*), many more oocytes may survive. *In Vitro* Matuation (IVM) is a method in which immature oocytes in antral follicles will be extracted and mature in laboratory conditions. There are many clinical benefits in this respect, despite of some pitfalls. The number of collected oocytes will increase by using external gonado-trophins in In Vitro Fertilization (IVF). However, these powerful medications increase the chance of ovarian hyperstimulation syndrome (OHSS). IVM reduces the amount of these external gonado-trophins and subsequent drastic effects.

IVM can be categorized into two groups: unstimulated cycles, and stimulated intracyto-plasmic sperm injection (ICSI) cycles. In unsti-mulated cycles, there is no external gonadotrophin administration, which may be beneficial for avoiding ovarian hyperstimulation syndrome (OHSS).

About 15-20% of harvested oocytes in IVF cycles are immature and in some cases, germinal vesicle (GV) oocytes may include a larger part of retrieved oocytes (1). These liberated immature oocytes may have developmental competence. IVM culturing is an opportunity to preserve these potentially good oocytes.

The in vitro maturation (IVM) was first proposed in 1935 and subsequentlty in 1969. The first birth occurred in 1991 by oocyte donation and in 1994 with own oocytes in PCO & PCOs patients (2-5). Almost 1200 IVM babies have been born internationally by using this technique.

Providing a culture system that is similar to follicle environment and producing oocytes resulting in healthy baby is the most important challenge in IVM. Oocytes grow and mature from primordial follicle to preovulatory stage through a long period and complex process. The normal diameter of the *in vivo* matured human oocyte is 110–120 µm. During folliculogenesis, the diameter of oocyte reaches from 30 µm in primordial follicle up to 120 µm at the time of antrum formation (6). Cavilla et al. (7) measured the diameter of collected immature oocytes before and after IVM culture and confirmed the size dependence of maturation.

The size of follicles may affect the subsequent embryonic development. The oocytes have more competencies in larger follicles. The minimum size of follicle required for developmental competence in humans is estimated to 5-7 mm in diameter (8). There are evidences demonstrating that the competency of oocyte is determined before antrum formation. Good quality oocytes force follicular growth which is influenced by circumstances of hormonal status up to ovulation. The atresia of follicles in early stage might be due to low quality oocytes (9).

There are some reports that indicate signifi-cant improvements in IVM rate by human chorionic gonadotrophin (hCG) priming before the retrieval of immature oocytes from PCOs patients (10-12). Improvement of IVM in PCO and PCOs and some other cases has led to planning of IVM protocol by some authors. In stimulated ICSI cycles, immature oocytes are cultured for 24-30 h. Extrusion of the polar body indicates that oocyte can be injected for further culturing. Acceptable number of such IVM produced embryos in early cleavage stage will be available by using this method (1). In our study of factors affecting IVF (13) and ICSI (14) outcome, the quality of oocytes and number of four cell embryos appeared to have significant predictive value. Although there are dissimilarities in fertilization and cleavage rate in different IVM studies (Table 1), in our previous study, they were not significantly different in IVM and *in vivo *MII oocytes (1). It seems that the value of four cells embryo transfer in pregnancy rate of ICSI cycles may have the same predictive value in IVM cycles. However, IVF centers have not adopted IVM widely. Many centers prefer the stimulated protocols even in typical cases. It is probably due to the fact that IVM protocol is not generally accepted as a reliable and valuable method. Nevertheless, the occurrence of pregnancies and delivered babies by using immature oocytes could not be ignored and might be a strong evidence for developmental competence of immature oocytes in particular conditions. It seems that the main question is that in which conditions we can obtain proper immature oocytes for IVM. This review is based on literature reports and the author’s experience. It has been attempted to provide the latest knowledge in IVM and highlight the most important related factors.

**IVM patients**

The main reason for IVM is definitely related to avoid external gonadotrophin administration and prevention of OHSS. Women with PCO and PCOs that have greater number of antral follicles form the main group in IVM (15-16). Egg donors, and women with infertility solely due to male factors or those who have had tubal ligation could reasonably be offered IVM (17). It has been reported that immature oocytes collected from women with regular menstrual cycles have the capacity to mature, fertilize and produce embryos leading to pregnancy (18).

In a recent study, in women with regular menstrual cycles and normal appearing ovaries that were recruited in unstimulated IVF cycles combined with IVM, the retrieval rate of mature oocytes was 62.5% per cycle, while immature cumulus oocyte complex (COC) was collected in 74/80 (92.5%) cycles. Embryo transfer was significantly increased with addition of IVM transferable embryos while live birth was not significantly different (19). Women with a previous poor response to IVF might be another indication for subsequent IVM. In a case control study IVM compared with IVF in PCOs patients, while there were 11.2% OHSS in IVF group and none in IVM, the pregnancy rate was not significantly different between the two groups (20).

There is a pitfall in stimulated cycles specifically in conditions that the percentage of immature oocytes is high in spite of mature oocytes (MII) or when GV and MI oocytes are the only available oocytes. Extensive studies are focused on these left over oocytes (1, 21- 23). In our study, a total of 279 oocytes were collected from 26 ICSI candidate women aged 18-37 years. The rates of maturation, fertilization and cleavage in IVM oocytes were compared to *in vivo *matured sibling oocytes. Our study showed that culturing GV oocytes can produce acceptable numbers of embryos in early stage of development. It seems that IVM in ICSI stimulated cycles can be conditioned at least in situations that the risk of embryo transfer cancellation exist.

**Follicular size related to oocyte maturity**

A female newborn has approximately 1-2 million oocytes which are surrounded by a flat single layer of squamous cells, called primordial follicles. Primary oocytes, approximately 30 µm in diameter, constitute most of follicles in women of all ages. Follicle growth from the primordial stage takes more than 2 months. During this long period, with increasing follicular diameter, the oocyte will attain developmental competence to be fertilized and undergo embryogenesis. The human oocyte gain of meiosis ability starts at the antral follicle, while its size reaches up to 100-120 µm. Theoretically, the antral follicles with diameter of 2-5 mm contain oocytes with nuclear and cytoplasmic competence. In practice, the minimum follicular diameter for producing competent oocyte is approximately 5 mm (24).

During folliculogenesis, oocytes contain messenger RNAs or proteins that are determinant in the early stage of embryonic development, before the activation of new created embryonic genome (9). These intracellular components might be established before antral formation and accumulate during follicle growth. The immature oocytes obtained from small size antral follicles (<6 mm) with acceptable pregnancy outcome (24), and *in vivo* matured oocytes collected from follicles (<10mm, 10-14mm) (25), might be good evidences in this respect.

In Guzman et al. report, IVM performed in PCOs and PCO patients that contained antral follicles <6mm with no priming hCG, after 40 h culture, 50.8% GV oocytes liberated polar body (24). The mean oocyte diameter significantly increased after maturation (mean diameter of resulted MII oocytes was 112.8 ± 3.7 µm). Clinical pregnancy rate was 5.4% in fresh embryo transfer (ET) while in delayed vitrified-warmed ET, it was 34.6%. The authors indicated that endometrial quality has a crucial role in pregnancy after non hCG-triggered IVM (24). In Son et al. study there were no significant differences in diameter of collected sibling *in vivo * matured oocytes, fertilized oocytes and good quality embryos on day 3 between the two groups of follicles <10mm and >10mm of PCO women (25). This study indicates that MII oocytes retrieved from small follicles have developmental competency.

It has been indicated that the synchronization of nuclear and cytoplasmic maturation is highly dependent on the timing and size of the follicle from which the oocytes are retrieved (17). However, the liberation of polar body does not mean that the ooplasm is mature. According to our experience, in some cases, GV oocytes that showed good ooplasm appearance and smooth feeling during injection after 24-30 h culture, usually fertilized in high rate and cleaved normally to produce early stage embryos. On the contrary, in other cases, GV oocytes that liberated polar body had dilute ooplasm and resulted in low fertilization rate and subsequent cleavage. In addition, follicles about 11mm in diameter in stimulated cycles (1), can contain competent oocytes capable to be promoted at least to early cleavage stages.

**The most important factors in developmental competency of oocytes**

Without doubt the quality of oocytes is the most important factor that determines IVM outcome. In this respect, intrinsic machinery of oocyte and extrinsic factors that influence it, should be noticed for better understanding and improvement of IVM.

**Cumulus oocyte morphological patterns**

The morphology of cumulus COC collected from follicles ≤12mm is different from those retrieved from mature follicles of standard IVF (Fig. 1). Immature oocytes rescued from smaller antral follicles are usually embedded in more compact cumulus cells (25). The number of dispersed CC increase with duration of hCG priming and growing of follicular size in IVM program (2). The patterns of COC are divided into three groups: dispersed, compacted and sparse. Dispersed CCs have an expanded CC and multiple layers of corona cells. GV oocyte that is completely masked with many layers of corona cells is considered as compacted CCs while very few coronal cells exist in sparse CCs. It had been indicated that COC with dispersed pattern was only experienced in hCG priming group (26). The comparison of different patterns of COCs showed that oocyte maturation

**Fig 1:**
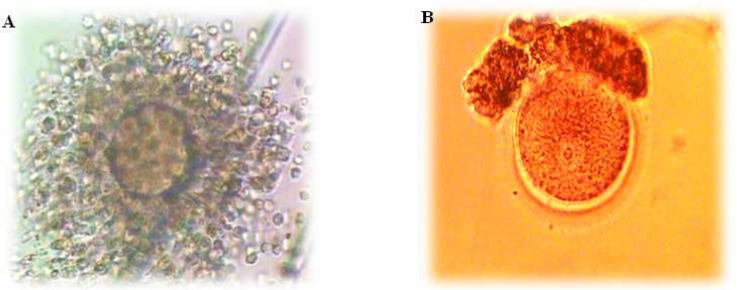
. A) Expanded COC collected from follicles >17 mm, and B) partially dissected GV oocyte collected in stimulated ICSI cycle

blastocyst formation significantly increase in COCs with dispersed cumulus cells (27). This study indicates that hCG primed IVM cycles of PCO(s) patients is correlated with their COCs pattern. Also, oocytes with dispersed cumulus complex obtained in hCG-primed IVM cycles without FSH administration had more developmental potential than those with other appearances (28). However, *in* *vivo* matured oocytes collected from small follicles (<10 mm) have developmental competency comparable to those collected from 10-14 mm (25). In general, larger follicles contain oocytes with more expanded corona radiata in the same patient. This study showed that follicular diameter is not related to embryo quality in *in* *vivo* matured oocytes in hCG-primed IVM cycles. It seems that hCG plays an essential role in attaining of components that are needed for competency in small size follicles.

**HCG administration**

The comparison of the rate of *in* *vivo* matured oocytes retrieved in IVM shows that the best time for administration of hCG is 35-38 h before egg collection, when the diameter of the largest follicle reaches 10-12 mm (29). Based on this report, increasing the interval between hCG priming and oocyte retrieval from 35 h to 38 h, leads to collecting more number of *in* *vivo* matured oocytes with dispersed CC pattern, elevating the IVM rate of immature oocytes and improving clinical outcome. However, clinical pregnancy was higher in *in* *vivo* derived embryos than in IVM.

**Oocyte diameter**

*In vivo* matured oocyte has a diameter ranging 112-119 µm. For GV oocyte in PCO(s), the mean diameter is around 107±1 µm while in ICSI patients mode, the diameter is 109-111 µm that is significantly larger than PCO(s) patients at collection time. The threshold diameter for IVM to MII is estimated 100 µm at collection and 103 µm on day 0 (7). In this study, 100% of *in vitro* matured oocytes collected from ICSI patients had diameters >106 µm. According to this report, maturation of human oocytes is size dependent and growing of oocytes during the final hours of *in vitro* maturation may be related to the following outcome. An increase of 3 µm average diameter leads to 8% increase in volume. Actually, the unnoticed increase in volume of oocytes during culture may provide valuable information about future competency. In addition, the growing of immature oocytes achieved from ICSI patients in the absence of somatic cells (denuded oocytes) shows that these oocytes do not need cumulus cells to increase their size.

**The diameter of dominant follicle**

Dominant follicles (DF) might have endocrine and paracrine harmful changing effects on remaining cohort of follicles. The diameter of DF has been determined to be at least 10 mm (30-31). Lim et al. (32) indicated that in natural IVF cycle combined with IVM, based on DF (12-14 mm), the best time to give hCG is 36 h before oocyte retrieval. In contrast, a recent report in combined natural IVF cycle with IVM showed that DF with mean diameter of 16.1 ± 0.128 mm on hCG day increased matured oocytes in overall from 35% to 53.8% with IVM oocytes (19). According to this report it seems that the size of DF in natural cycles does not have detrimental effects on remaining follicles.

As mentioned above, the oocytes with dispersed CC pattern produce better embryos and enhance pregnancy rate. Therefore, increased follicle diameter results in more dispersed CC pattern that may have predictive value of higher maturation rates and developmental competence.

**Culture medium for IVM and supplements**

Maturation of oocytes outside of the body needs to provide optimal environment similar to natural milieu inside the body. In this respect, maturation media has been prepared in different types usually supplemented with recombinant FSH and hCG. Patient-inactivated serum or follicular fluid has also been added as an exogenous protein source by some authors (19). Araujo et al. (33) indicated that TCM 199 is a better media than HTF medium in IVM of PCOs patients regarding maturation rate (82% vs. 56.9%), fertilization rate (70% vs. 39.4%), and embryo quality (81.3% vs. 41.7%). They added pyruvate and adenosine triphosphate (ATP) as essential supplementations for energy metabolites in IVM media. On the other hand, comparison of three IVM media in a mice model showed that there were no differences in developmental competency of immature oocytes between conventional IVM medium and blastocyst culture medium while TCM-199 was not a proper medium (34). These authors compared the formulation of different media and realized that taurine and calcium are unique components of blastocyst medium. In all species, there are large amounts of taurine and hypotaurine in gametes and in the embryo environment which is supposed to be antioxidant. Also, blastocyst culture media containing calcium lactate may have positive effect on mitochondrial function to attain sufficient ATP production. In contrast, Moschini et al. (35), showed that commercialized IVM media (including Medicult, Sage/Cooper Surgical, Sage IVF Inc and Cook), have no significant benefits to improve maturation of oocytes rescued from stimulated cycles compared with standard growth media.

It is well documented that Epidermal Growth Factor (EGF) stimulates mitosis and induces resumption of meiosis in oocytes in various mammals (36-38). Also, the supplementation of maturation medium with EGF and FSH improves significantly the nuclear and cytoplasmic maturation of sheep oocytes (39). However, the cumulus cells have an essential role in IVM and cannot be ignored even in the presence of EGF and FSH in porcine oocytes (40). In this study, in a serum - and hormone- free IVM medium, EGF had a synergistic effect with FSH on cytoplasmic maturation and only 15% to 20% of oocytes reached the MII stage in the absence of cumulus cells, FSH, and EGF.

Adding estradiol 17β (E2) to the IVM medium (TCM 199) was not necessary in sheep oocytes when the medium contained FF (39). Also, Kim et al. showed that while totaling E2 to IVM medium had no beneficial effect through IVM, E2 during the first half of the IVM period had positive effect in maturation rate and consequent embry-ogenesis (34).

In Funahashi et al. (41) experiments on porcine oocytes, glucose had an essential role in resumption of meiosis and cytoplasmic maturation was improved in association with pyruvate. They recommended the addition of Na pyruvate to a glucose containing, chemically defined medium for IVM. The developmental competence of oocytes had the lowest value (23%) in pyruvate only group and elevated in glucose only group (51%) while in the presence of glucose and pyruvate, more than 75% of oocytes matured to MII stage.

A recent study showed that supplementation of IVM medium with 2.0 µM resveratrol (3,4',5 -trihydroxystilbene), a naturally occurring phyto-alexin, a secondary plant metabolite, increases intracellular levels of glutathione (GSH) and decreases reactive oxygen species (ROS) during IVM of porcine oocytes and improves develop-mental competence of parthenogenetic and IVF embryos. The authors suggest that nature of the maturation medium significantly influences the expression of apoptosis-related genes in mature oocytes, cumulus cells, and blastocyst stage embryos, decreasing their susceptibility to apoptosis (42). In another study, Wu et al. (43) showed that adding 0.5 mg/mL of L-carnitine to IVM medium in porcine oocytes significantly increases blastocyst formation with less apoptosis after parthenogenesis activation, significantly reduces intracellular ROS and also elevates GSH concentrations. They inferred L-carnitine as an improving factor in developmental competence of porcine oocytes.

**Insemination methods and ICSI timing in IVM**

ICSI is a preferred method for insemination of IVM oocytes because of maturity of oocytes easily identified after denudation and IVF reduces fertilization chance after removal of cumulus cells. There are two methods to identify GV oocytes before removing the cumulus cells. In the sliding method, after the possible physical dissecting of cumulus cells, COC is sliding at the bottom of Petri dish to allow germinal vesicle observation. In the spreading method, COC are put into the Petri dish to spread. If no germinal vesicle is observed under stereomicroscope, CCs are removed to assess maturity.

Asynchronous maturation of the immature oocytes is an unfavorable feature that occurs during IVM. In an experiment, 43.2% of collected immature oocytes reached MII stage after 24 h. The denuded MI oocytes that liberated the first polar body by 32 h, were injected with intervals between 1-8 h. The fertilization rate was the same between the control group (oocytes that reached the MII stage after 24 h of *in vitro* culture) and others that had interval time of >1 h. The oocytes that were injected within 1 hour after extrusion of firstpolar body had 15.1% maturation only (44).Also, spindle location viewed by the Polscope was used as a tool to find out the appropriate ICSI timing in this study. All oocytes that were observed within 1 h after the first polar body (PB) liberation showed spindle location between cytoplasm and the first PB with the real maturational stage of telophase I. After 1 h of extrusion of the first PB, all oocytes were at real MII stage and had the spindle beneath the first PB. This means that IVM oocytes need at least 1 h to complete nuclear maturation after extrusion of first polar body.

**Women aging**

Fecundity declines by 35 years in women and diminishes remarkably after 40 years of age. Without using of any contraception, 30% of women at age 35 to 39 and 64% at age 40 to 44 remained childless. The age-related decline in fertility and live birth failure are attributable to oocyte abnormality. So,aneuploidy is a major cause of spontaneous abortion in aged women. The ovarian reserve shows fertility potential in female regarding the number and quality of oocytes. In this respect, the measurement of basal FSH and estradiol on day 3 of menstrual cycle, that is determined <10 mIU/ml and <80 pg/ml for FSH and estradiol respectively, are routine tests for ovarian reserve. Also, the number of small antral follicles visible in ultrasound correlates with ovarian reserve. However, female aging and ovarian reserve could be independent factors in the prediction of fertility and should be considered when counseling infertile couples. In most groups, ovarian response has been determined according to the number of retrieved oocytes and it affects pregnancy rate more than two times compared to age independent manner. It is indicated that ovarian response together with ovarian reserve can predict age-independent IVF outcome (45). With the rising of maternal age, the ovarian reserve decreases. Spandorfer et al. (46), showed that although IVF is a reasonable choice for very advanced aged women (45 years of age), only those with retrieved oocytes >5 had a delivery. In a recent study, women in different ages were analyzed in IVM program. The rates of clinical pregnancy in 20–25, 26–35 and 36–39 years were 42.1%, 33.8% and 35.1%, respectively. No clinical pregnancy happened in patients more than 40 years of age. It is concluded that women over 40 years are poor candidates for IVM treatment (47).

The aneuploidy in human oocytes increases with age and elevates from 29% to 56% in 20-34 and 34-45 years, respectively (48). Clinically, the FSH level in follicular fluid in stimulated cycles ranged from 5.54 to 7.80 IU/L, which is 1–2 IU/L lower than the serum FSH level. A recent study showed that high FSH concentration resulted in aneuploidy in IVM of human oocytes (49). Therefore, high dose of gonadotrophins should be used with caution in IVM.

**Clinical outcome in IVM**

The clinical outcome of IVM is indicated by fertilization, cleavage, clinical pregnancy and live birth. However, it should be noticed that IVM resulting in stimulated and unstimulated cycles are basically different. 

Depending on some factors and conditions mentioned above, IVM outcome might be affected and therefore it is not unexpected that the results are dissimilar in different studies. The outcome of IVM cycles from literature are summarized in table 1. 

## Conclusion

Oocyte maturation is associated to follicular size. So the administration of hCG is determined by the largest follicle diameter when it reaches 10-12 mm. It has been indicated that increasing the time of hCG injection up to 38 h before oocyte retrieval will result in collecting more *in vivo* matured oocytes with dispersed CC pattern, increasing IVM rate and improving clinical outcome. The oocytes obtained from follicles of patients have different sizes. It is reported that oocyte has to have a diameter of 100 µm at collection and 103 µm on day 0 to develop MII phase. It seems that growing of oocytes during the final hours of *in vitro* maturation has profound effect on the outcome.

**Table 1:** Outcome of IVM cycles from literature

Authors (year)	No. of GV oocytes	*In vitro* maturation rate (%)	Fertilization rate (%)	Cleavage rate (%)	Clinical pregnancy rate/embryo transfer (%)	Live birth
Nagy, 1996 (20)	14	64	78	71	100	1
Kim et al., 2000 (50)	168	66.7	51.8	84.5	-	-
Yoon et al., 2001 (17)	506	74.3	72.6	89	17.6	9
Hyun et al., 2007 (42)	901	61.15	78.4	Good Quality: 39.8	-	-
Lim et al., 2007 (30)	619	80	75	96	35% (mixed with *in vivo* matured oocytes)	-
Shu et al., 2007 (49)	27.1	37.5	44.2	100	2	-
Son et al., 2008 (50)	1086	65.1	71.7	84.4	38.7	7ongoing pregnancy, 13 delivery
Son et al., 2008 (27)	336	46.3	79.8	95.5	40.9 % (mixed with *in vivo* matured oocytes)	
Araujo et al., 2009 (31)	61	82	70	91.4	2	-
Reichman et al., 2010 (51)	37.2	35.1	60.0	20.5	None	none
Escrich et al., 2011 (22)	131	74.8	59.7	-	-	-
Wiser et al., 2011 (45)		58	68.5	-	33.8	30%
Guzman et al., 2012 (22)	967	50.8	63.7	Good Quality:45.4	34.6 (after warming)	3 delivery, 8 ongoing
Tang-Pedersen et al., 2012 (18)	253	33.2	58.3	18.75	6.7	1

Dominant follicle has detrimental effect on remaining follicles when it exceeds the diameter of 12-14 mm. Allowing follicles to grow up more is desirable because more oocytes with dispersed CC pattern and subsequently better embryos will be achieved. In a recent report DF with mean diameter of 16.1 ± 0.128 mm on hCG day increased significantly harvested matured oocytes in natural IVF cycle combined with IVM.

There are different types of maturation media that are generally supplemented with recombinant FSH and hCG. Pyruvate and adenosine triphosphate (ATP) as energy metabolites, taurine and calcium lactate as an antioxidant and enhancer of the mitochondrial function are branded. However, Epidermal Growth Factor (EGF) that stimulates mitosis and induces resumption of meiosis in oocytes in various mammals, is not used commonly in IVM. Also, estradiol 17β (E2) during the first half of the IVM period, glucose in association with pyruvate and L-carnitine are recommended as IVM additive in animal models.

The injection of IVM oocytes must be delayed at least 1 h after extrusion of the first polar body. In addition, it is indicated that in stimulated ICSI cycles denudation performed after 3 h incubation resulted in significantly improved fertilization and implantation rate.

IVM is not recommended for women over 40 years. The ovarian reserve that is reflected by basal FSH and estradiol on day 3 of menstrual cycle and female aging can be the independent factors in the prediction of fertility. Also, ovarian response reflected by the number of retrieved oocytes significantly affects pregnancy rate. High FSH concentration resulted in aneuploidy in IVM of human oocytes and therefore should be used with caution in IVM.

Over 1200 babies have already been born internationally by using IVM technique. Avoiding of external gonadotrophin administration and prevention of OHSS are the main advantages of IVM. Medications do not work in the same manner in all cases, so the number and quality of oocytes are adversely affected and the patients may encounter unfortunate results. This situation is not in favor of IVF team and will encourage accepting IVM more widely in the future. However, PCO, and PCOs are the main and reasonable groups in IVM indication.

## References

[1] Farsi MM, Jorsaraei SG, Esmaelzadeh S (2011). In vitro maturation of germinal vesicle oocytes in stimulated intracytoplasmic sperm injection cycles. Cell J.

[2] Pincus G, Enzmann EV (1935). The Comparative Behavior of Mammalian Eggs in vivo and in vitro : I. The Activation of Ovarian Eggs. J Exp Med.

[3] Edwards RG, Bavister BD, Steptoe PC (1969). Early stages of fertilization in vitro of human oocytes matured in vitro. Nature.

[4] Cha KY, Koo JJ, Ko JJ (1991). Pregnancy after in vitro fertilization of human follicular oocytes collected from nonstimulated cycles, their culture in vitro and their transfer in a donor oocyte program. Fertil Steril.

[5] Trounson A, Wood C, Kausche A (1994). In vitro maturation and the fertilization and developmental competence of oocytes recovered from untreated polycystic ovarian patients. Fertil Steril.

[6] Gosden RG, Grudzinskas JG, Yovich JL (1995). Ovulation 1: oocyte development throughout life. Gametes-The Oocyte.

[7] Cavilla JL, Kennedy CR, Byskov AG (2008). Human immature oocytes grow during culture for IVM. Hum Reprod.

[8] Trounson A, Anderiesz C, Jones G (2001). Maturation of human oocytes in vitro and their developmental competence. Reproduction.

[9] Mermillod P, Dalbies - Tran R, Uzbekova S (2008). Factors Affecting oocyte quality: who is driving the follicle? Reprod Domest Anim.

[10] Chian RC, Buckett WM, Tulandi T (2000). Prospective randomized study of human chorionic gonadotrophin priming before immature oocyte retrieval from unstimulated women with polycystic ovarian syndrome. Hum Reprod.

[11] Lin YH, Hwang JL, Huang LW (2003). Combination of FSH priming and hCG priming for in-vitro maturation of human oocytes. Hum Reprod.

[12] Son WY, Yoon SH, Lim JH (2006). Effect of gonadotrophin priming on in-vitro maturation of oocytes collected from women at risk of OHSS. Reprod Biomed Online.

[13] Esmailzadeh S, Faramarzi M, Farsi M (2008). The predictors of clinical fertilization in IVF cycles. Acta Med Iran.

[14] Farsi M, Jorsaraei A, Hajiahmadi M (2007). Role of embryo morphology in Intracytoplasmic Sperm Injection cycles for prediction of pregnancy. IJRM.

[15] Chian RC, Lim JH, Tan SL (2004). State of the art in in-vitro oocyte maturation. Curr Opin Obstet Gynecol.

[16] Jurema MW, Nogueira D (2006). In vitro maturation of human oocytes for assisted reproduction. Fertil Steril.

[17] Yoon HG, Yoon SH, Son WY (2001). Pregnancies resulting from in vitro matured oocytes collected from women with regular menstrual cycle. J Assist Reprod Genet.

[18] Tang-Pedersen M, Westergaard LG, Erb K (2012). Combination of IVF and IVM in naturally cycling women. Reprod Biomed Online.

[19] Child TJ, Phillips SJ, Abdul-Jalil AK (2002). A comparison of in vitro maturation and in vitro fertilization for women with polycystic ovaries. Obstet Gynecol.

[20] Nagy ZP, Cecile J, Liu J (1996). Pregnancy and birth after intracytoplasmic sperm injection of in vitro matured germinal-vesicle stage oocytes: case report. Fertil Steril.

[21] Chian RC, Tan SL (2002). Maturational and developmental competence of cumulus-free immature human oocytes derived from stimulated and intracytoplasmic sperm injection cycles. Reprod Biomed Online.

[22] Escrich L, Grau N, Mercader A (2011). Spontaneous in vitro maturation and artificial activation of human germinal vesicle oocytes recovered from stimulated cycles. J Assist Reprod Genet.

[23] Guzman L, Ortega-Hrepich C, Albuz FK (2012). Develop-mental capacity of in vitro-matured human oocytes retrieved from polycystic ovary syndrome ovaries containing no follicles larger than 6 mm. Fertil Steril.

[24] Son WY, Chung JT, Dahan M (2011). Comparison of fertilization and embryonic development in sibling in vivo matured oocytes retrieved from different sizes follicles from in vitro maturation cycles. J Assist Reprod Genet.

[25] Yang SH, Son WY, Yoon SH (2005). Correlation between in vitro maturation and expression of LH receptor in cumulus cells of the oocytes collected from PCOS patients in HCG-primed IVM cycles. Hum Reprod.

[26] Son WY, Tan SL (2010). Laboratory and embryological aspects of hCG-primed in vitro maturation cycles for patients with polycystic ovaries. Hum Reprod Update.

[27] Son WY, Chung JT, Chian RC (2008). A 38 h interval between hCG priming and oocyte retrieval increases in vivo and in vitro oocyte maturation rate in programmed IVM cycles. Hum Reprod.

[28] Pache TD, Wladimiroff JW, de Jong FH (1990). Growth patterns of nondominant ovarian follicles during the normal menstrual cycle. Fertil Steril.

[29] Fauser BC, Van Heusden AM (1997). Manipulation of human ovarian function: physiological concepts and clinical consequences. Endocr Rev.

[30] Lim JH, Yang SH, Chian RC (2007). New alternative to infertility treatment for women without ovarian stimulation. Reprod Biomed Online.

[31] de Araujo CH, Nogueira D, de Araujo MC (2009). Supplemented tissue culture medium 199 is a better medium for in vitro maturation of oocytes from women with polycystic ovary syndrome women than human tubal fluid. Fertil Steril.

[32] Kim M, Hong SJ, Lee JH (2011). Comparison of in vitro maturation media of immature oocytes: the effectiveness of blastocyst culture media. Fertil Steril.

[33] Moschini RM, Chuang L, Poleshchuk F (2011). Commercially available enhanced in vitro maturation medium does not improve maturation of germinal vesicle and metaphase I oocytes in standard in vitro fertilization cases. Fertil Steril.

[34] Das K, Stout LE, Hensleigh HC (1991). Direct positive effect of epidermal growth factor on the cytoplasmic maturation of mouse and human oocytes. Fertil Steril.

[35] Bolamba D, Russ KD, Harper SA (2006). Effects of epidermal growth factor and hormones on granulosa expansion and nuclear maturation of dog oocytes in vitro. Theriogenology.

[36] Lindbloom SM, Farmerie TA, Clay CM (2008). Potential involvement of EGF-like growth factors and phosphodiesterases in initiation of equine oocyte maturation. Anim Reprod Sci.

[37] Guler A, Poulin N, Mermillod P (2000). Effect of growth factors, EGF and IGF-I, and estradiol on in vitro maturation of sheep oocytes. Theriogenology.

[38] Uhm SJ, Gupta MK, Yang JH (2010). Epidermal growth factor can be used in lieu of follicle-stimulating hormone for nuclear maturation of porcine oocytes in vitro. Theriogenology.

[39] Funahashi H, Koike T, Sakai R (2008). Effect of glucose and pyruvate on nuclear and cytoplasmic maturation of porcine oocytes in a chemically defined medium. Theriogenology.

[40] Kwak SS, Cheong SA, Jeon Y (2012). The effects of resveratrol on porcine oocyte in vitro maturation and subsequent embryonic development after parthenogenetic activation and in vitro fertilization. Theriogenology.

[41] Wu GQ, Jia BY, Li JJ (2011). L-carnitine enhances oocyte maturation and development of parthenogenetic embryos in pigs. Theriogenology.

[42] Hyun CS, Cha JH, Son WY (2007). Optimal ICSI timing after the first polar body extrusion in in vitro matured human oocytes. Hum Reprod.

[43] Yih MC, Spandorfer SD, Rosenwaks Z (2005). Egg production predicts a doubling of in vitro fertilization pregnancy rates even within defined age and ovarian reserve categories. Fertil Steril.

[44] Spandorfer SD, Bendikson K, Dragisic K (2007). Outcome of in vitro fertilization in women 45 years and older who use autologous oocytes. Fertil Steril.

[45] Wiser A, Son WY, Shalom-Paz E (How old is too old for in vitro maturation (IVM) treatment? Eur J Obstet Gynecol Reprod Biol 2011).

[46] Sandalinas M, Marquez C, Munne S (2002). Spectral karyotyping of fresh, non-inseminated oocytes. Mol Hum Reprod.

[47] Xu YW, Peng YT, Wang B (2011). High follicle-stimulating hormone increases aneuploidy in human oocytes matured in vitro. Fertil Steril.

[48] Kim BK, Lee SC, Kim KJ (2000). In vitro maturation, fertilization, and development of human germinal vesicle oocytes collected from stimulated cycles. Fertil Steril.

[49] Shu Y, Gebhardt J, Watt J (2007). Fertilization, embryo development, and clinical outcome of immature oocytes from stimulated intracytoplasmic sperm injection cycles. Fertil Steril.

[50] Son WY, Chung JT, Herrero B (2008). Selection of the optimal day for oocyte retrieval based on the diameter of the dominant follicle in hCG-primed in vitro maturation cycles. Hum Reprod.

[51] Reichman DE, Politch J, Ginsburg ES (2010). Extended in vitro maturation of immature oocytes from stimulated cycles: an analysis of fertilization potential, embryo develop-ment, and reproductive outcomes. J Assist Reprod Genet.

